# Nearest-neighbour clusters as a novel technique for assessing group associations

**DOI:** 10.1098/rsos.140232

**Published:** 2015-01-21

**Authors:** Sean A. Rands

**Affiliations:** School of Biological Sciences, University of Bristol, Bristol Life Sciences Building, 24 Tyndall Avenue, Bristol BS8 1TQ, UK

**Keywords:** social networks, hierarchies, behavioural ecology, social behaviour

## Abstract

When all the individuals in a social group can be easily identified, one of the simplest measures of social interaction that can be recorded is nearest-neighbour identity. Many field studies use sequential scan samples of groups to build up association metrics using these nearest-neighbour identities. Here, I describe a simple technique for identifying clusters of associated individuals within groups that uses nearest-neighbour identity data. Using computer-generated datasets with known associations, I demonstrate that this clustering technique can be used to build data suitable for association metrics, and that it can generate comparable metrics to raw nearest-neighbour data, but with much less initial data. This technique could therefore be of use where it is difficult to generate large datasets. Other situations where the technique would be useful are discussed.

## 2. Introduction

In order to understand the evolution and ecology of social behaviour, we must first observe and quantify the interactions between members of socially connected groups. Once we have information at this basic level of interaction, we can then begin to build networks and test hypotheses regarding their structure [1–3]. Different information can be collected about interactions between individuals, with the most basic observational information being information about spatial proximity. If a dataset is constructed using multiple observations about spatial proximity, metrics such as association measures [4] can then be constructed.

When individuals within a group can be easily identified, field studies typically use either focal sampling, where pre-selected individuals are followed for a given length of time, collecting sequential metrics about their associations with other individuals, or scan sampling, where the associations of all measurable individuals are recorded at a given moment [5]. Both techniques have their merits for recording different aspects of social behaviour, but I focus on cases where scan sampling is conducted, which arguably gives a more reliable measure of associations when some individuals in the group are unlikely to interact with others (and therefore may be largely missing from a dataset when they are not the focal subject during focal sampling).

**Figure 1.**
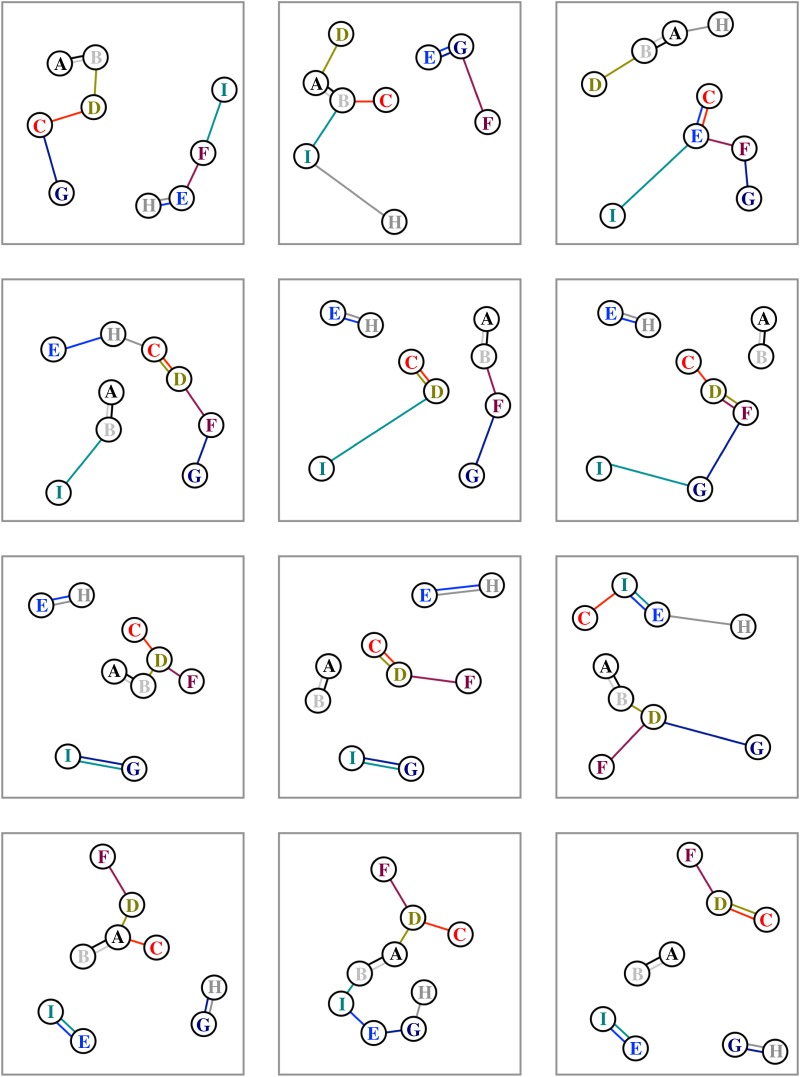
An illustration of group association behaviour, considered over 12 observations. Lines represent the nearest-neighbour associations recorded.

Scan sampling of all individuals can give a quick measurement of intragroup association in the field, forcing a record to be taken for all individuals. The simplest association metric involves identifying the nearest neighbour of each individual. This is a fast and reliable technique that is frequently implemented in studies of primates [6–8] and herding ungulates [9,10]. For example, figure 1 gives 12 separate observations of the spatial proximities of nine identified individuals in a group, where coloured lines connect each individual to their closest neighbour. Over multiple observations, the nearest-neighbour count matrix that is generated is unlikely to be symmetric, as the closest neighbour to a focal individual may itself be closer to a different individual (for example, in the top left panel of figure 1, the closest neighbour of D is B, but B's closest neighbour is A, and not D). Table 1 gives a nearest-neighbour count matrix calculated for the 12 observations in figure 1. Typically, these count matrices are then analysed to generate various association metrics [1,4].

**Table 1.** Nearest-neighbour count matrix, showing the number of times each member of a group recorded over 12 observations (figure 1) was the nearest neighbour of a given focal individual.

		nearest-neighbour identity								
		A	B	C	D	E	F	G	H	I
focal individual	A	—	12	0	0	0	0	0	0	0
	B	12	—	0	0	0	0	0	0	0
	C	1	1	—	8	1	0	0	0	1
	D	3	4	4	—	0	1	0	0	0
	E	0	0	1	0	—	0	1	6	4
	F	0	1	0	8	2	—	1	0	0
	G	0	0	1	1	2	4	—	2	2
	H	1	0	1	0	6	0	3	—	1
	I	0	3	0	1	4	1	3	0	—

Recording nearest-neighbour metrics is extremely simple to implement in the field if all individuals are identifiable, but using them to generate a simple nearest-neighbour count matrix means that some information about proximity is lost: considering only the nearest neighbour loses some of the information about close multi-individual associations within the group. For example, individuals within foraging groups of chacma baboons, *Papio ursinus*, tend to cluster, so that each individual is within 5 m of a nearest neighbour [11], meaning that although a large group may seem dispersed, all individuals are potentially closely connected to all the other individuals via a diffuse network of nearest-neighbour connections. These large groups may not be visible within a dataset as different individuals in a group are more or less likely to be strongly associated with other individuals, through diverse processes such as mate guarding, infant care and social hierarchies. Figure 1 gives a particularly strong example, where individuals A and B are assumed to be very tightly bonded: for example, we could assume that A is a mother tending to a dependent infant B that maintains close proximity to her at all times. Tight, close proximity relationships such as these are likely to distort how the relationships of other individuals in the group are associated with these tightly connected individuals. For example, individuals C and D could be older infants that maintain close proximity to their mother A, but spend some time ranging through the group and interacting with other individuals. Although A may be giving attention to C and D, her closer proximity to B will mean that her relationship with C and D will be much less obvious within a nearest-neighbour count, as can be observed in table 1. If we take multiple observations, we begin to piece together these more distant relationships between individuals, but this will depend upon the amount of data that we collect, and may be difficult if the group being studied is only visible for short windows of time.

In this paper, I describe an extra layer of analysis that gives us a means of aggregating relationships between individuals faster, by identifying individuals who are members of a nearest-neighbour cluster, following the definition used by Hamilton [12]. As well as providing a different measure for assessing grouping relationships between identifiable individuals, this technique gives a faster means of identifying associations within groups.

## 3. Methods

### 3.1 Local group association

Hamilton [12] describes a nearest-neighbour cluster as a grouping that contains all the individuals that are the nearest individual to at least one other member of the cluster (see references [13,14] for an implementation of clustering). The smallest nearest-neighbour cluster could therefore be two individuals who share each other as their nearest neighbours, as can be seen in the bottom right panel of figure 1 where A and B, E and I, and G and H are three separate two-individual clusters. The largest possible cluster will consist of all the members of the visible group, as can be seen in the bottom middle panel of figure 1.

It is not necessary to record clusters *in situ*, as nearest-neighbour clusters can be constructed for a given moment if the identities of each individual's closest neighbour are known—this is a relatively straightforward form-filling task in the field, and only requires a little extra computation by hand during analysis. Having identified all the clusters within the group, a tally needs to be made of which other individuals a focal shares its group with. For example, in the top left panel of figure 1, individuals A, B, C, D and G should each be scored as being in a cluster with each other, and the same should be done for E, F, H and I. Tallying shared cluster membership over all the observations made gives a local group association matrix: table 2 gives the matrix for the 12 observations given in figure 1. Note that unlike the nearest-neighbour count matrix, the local group association matrix is symmetrical, as there is no directionality implied by assuming group memberships.

**Table 2.** Local group cluster matrix, constructed from the 12 observations given in figure 1.

		nearest-neighbour identity								
		A	B	C	D	E	F	G	H	I
focal individual	A	—	12	5	7	1	5	4	3	3
	B		—	5	7	1	5	4	3	3
	C			—	10	4	8	5	4	6
	D				—	2	8	5	4	4
	E					—	5	4	8	6
	F						—	7	3	4
	G							—	4	5
	H								—	4
	I									—

### 3.2 Testing the techniques

To test the performance of the local group association technique against the established nearest-neighbour count technique, I created three datasets, using NetLogo v. 5.0.5 [15] to simulate the movement of individuals with known associations to generate a series of sequential observations. In each, 25 individuals moved through the environment, and nearest-neighbour identities for each individual were recorded at defined intervals. A sequential linear social hierarchy was imposed on the individuals in two of these simulations, where each individual showed a probability of being attracted towards those individuals closest to it within the hierarchy (either the individuals immediately above and below it, termed *most similar hierarchy attraction*, or those two above or below, termed *less similar hierarchy attraction*). Another set of simulations considered the case where social attraction was not based on any hierarchy, which should consequently give a random association matrix as the identity of closest neighbours is determined purely by an individual's drift through its social environment (termed *random choice*). Appendix A describes the models in detail.

Each of the three models generated a series of 10 000 sequential nearest-neighbour associations, which were then converted to nearest-neighbour count and local group association matrices using a piece of C++ code (see the electronic supplementary material), and compared using the metrics described in §3.3. Each model was run 100 times: for all the statistics collected, a mean and standard deviation across the 100 simulations was calculated.

### 3.3 Comparing the two techniques

The metric I describe assumes that multiple sequential observations have been collected. For observation *n*, I define *s*_*i*,*j*,*n*_ as the number of observations (up to and including observation *n*) where individual *i* was the closest neighbour to individual *j* (where we assume that *i* and *j* are different individuals), and *g*_*i*,*j*,*n*_ as the number of observations where *i* and *j* were in the same nearest-neighbour cluster: corresponding to the individual entries in the nearest-neighbour count and local group association matrices, respectively. I can then calculate the overall difference at observation *n* between the cumulative matrices of nearest-neighbour identity counts and of nearest-neighbour cluster counts using

$$d_{n}=\sum_{i,j}{\left(\frac{s_{i,j,n}}{S_{n}}-\frac{g_{i,j,n}}{G_{n}}\right)}^{2},$$

where the two matrices were standardized beforehand using $S_{n}=\sum_{i,j}s_{i,j,n}$ and $G_{n}=\sum_{i,j}g_{i,j,n}$. It should be noted that although there is double-accounting in the *g*_*i*,*j*,*n*_ term (as *g*_*i*,*j*,*n*_=*g*_*j*,*i*,*n*_), this is controlled for by standardizing with the *S*_*n*_ and *G*_*n*_ terms. *d*_*n*_ can potentially take a value equal to or greater than 0 (and less than or equal to 1, which would be extremely unlikely): larger values indicate a greater dissimilarity between the two cumulative count matrices considered here.

I also examined how quickly the two different matrices changed as the amount of data collected increased, by comparing the matrices generated from a given amount of data (from the first *m* observations recorded) with matrices that included an additional quantity of data, from the first *n* datapoints (where *m*<*n*). These were calculated as

$${\text{cluster}}_{n,m}=\sum_{i,j}{\left(\frac{g_{i,j,n}}{G_{n}}-\frac{g_{i,j,m}}{G_{m}}\right)}^{2}$$

and

$${\text{identity}}_{n,m}=\sum_{i,j}{\left(\frac{s_{i,j,n}}{S_{n}}-\frac{s_{i,j,m}}{S_{m}}\right)}^{2},$$

where the cluster_*n*,*m*_ compares a standardized cumulative nearest-neighbour cluster count at observation *n* with that taken at observation *m* (where we assume *m*<*n*), and count_*n*,*m*_ does the same for the cumulative nearest-neighbour identity count.

For all the simulations, *d*_*n*_, cluster_*n*,*m*_ and identity_*n*,*m*_ were calculated for *n*=(1,2,…,10 000) and *m*=(1,10,20,40,80).

## 4. Results

**Figure 2.**
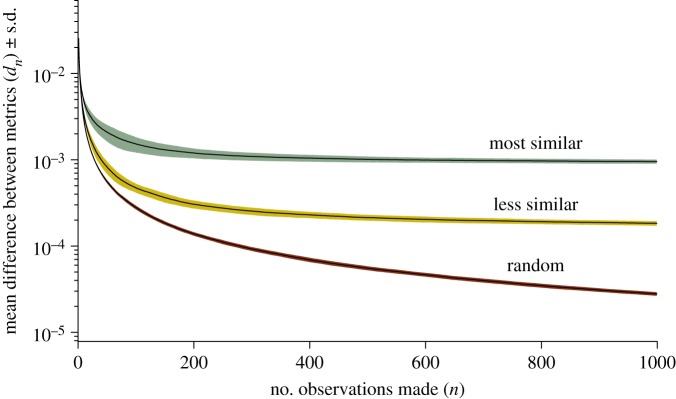
Differences between the two metrics taken, for observations taken from the three models described (from top to bottom: most similar hierarchy attraction, less similar hierarchy attraction and random attraction).

The matrices generated using both nearest-neighbour counts and nearest-neighbour metrics become more similar as the number of observations used to generate them increases. Although the three different behavioural models considered led to differing levels of similarity for a given number of observations, figure 2 demonstrates that the difference between the two measures will tend towards an asymptotic value, which is unsurprising as they are not independent measures.

**Figure 3.**
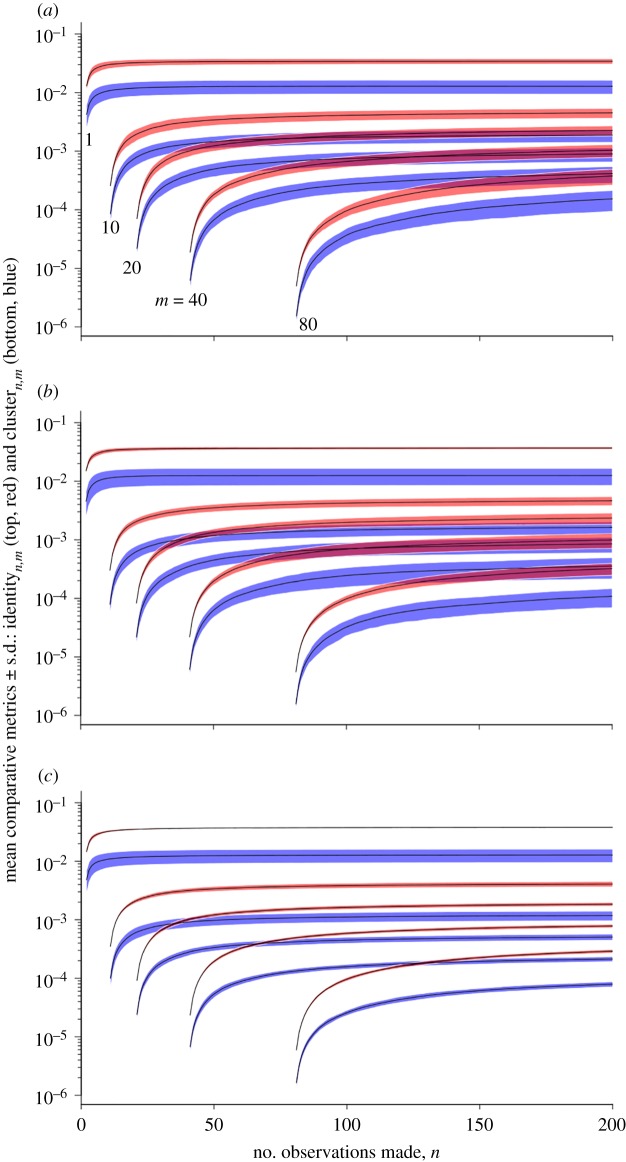
Comparing the performance of the two metrics taken, dependent upon the number of observations used. Pairs represent the metrics for differing values of *m*, with red (top) lines comparing nearest-neighbour count matrices (identity_*n*,*m*_) and blue (bottom) lines comparing nearest-neighbour cluster matrices (cluster_*n*,*m*_). The three panels correspond to results from the three models considered: (*a*) most similar hierarchy attraction; (*b*) less similar hierarchy attraction and (*c*) random attraction.

The cluster_*n*,*m*_ and identity_*n*,*m*_ metrics show the amount of variation within both the nearest-neighbour cluster and nearest-neighbour count association metrics when the number of observations used to generate them is increased. As the value of *m* is increased in figure 3, there is an overall reduction in the values of cluster_*n*,*m*_ and identity_*n*,*m*_ that are generated, meaning that subsequent association matrices become more similar as the number of observations used to generate them is increased (echoing the results in figure 2). As the number of observations increased, the nearest-neighbour cluster metric stabilized much faster, as demonstrated by the lower values of cluster_*n*,*m*_ when compared with the corresponding identity_*n*,*m*_.

## 5. Discussion

The nearest-neighbour cluster metric developed in this paper requires few observations to give a stable association matrix, when compared with the established nearest-neighbour count metric. This suggests that the metric will be useful in systems where it is difficult to collect data, such as in field studies with animals that are difficult to observe (such as through having wide ranges, or living in complex environments where measurements can only be made when the group is visible). Reducing the number of observations required to gain a meaningful association metric also means that more can be done with larger datasets, such as making it easier to compare how social behaviour networks change over time or in response to perturbation [3,16].

Although the technique described is motivated for raw data consisting of the identities of the *spatially* nearest individual to all group members, it is possible to use the technique with other forms of data. Where the physical positions in space of individuals in a group have been recorded using biotelemetry techniques [such as [17–20]], it is straightforward to reconstruct nearest-neighbour relationships for all individuals recorded (as figure 1 demonstrates), although it should be acknowledged that data with accurate physical positions of all individuals may yield very different relationship metrics if absolute distances between all individuals are used (so in figure 1, individual C is in the same cluster as F eight times, and in the same cluster as A five times, but is physically closer to A more times than it is to F), suggesting that researchers should be careful in deciding which summary statistic is likely to give the most meaningful interpretation of their data if exact physical distances can be obtained. Temporal proximity could also be used: if animals have to pass through a specific space like a known bottleneck or open space, their passage order can be recorded sequentially (such as in the movement of black-and-white snub-nosed monkeys, *Rhinopithecus bieti*, across forest gullies recorded in Neisen *et al*. [20]), with the order of passage through the space being used to construct the association metric. Both physical position and temporal passage through a single space are techniques that could generate meaningful association data if done remotely (but of course may already yield other useful association metrics, which could be compared with the clustering technique used here).

The method I describe relies on data being collected for all the individuals in a group during a sample period, rather than something more similar to focal sampling (such as in references [11,21–25]), where the data are focused on recording the neighbours of one or several focal individuals at a moment in time, therefore potentially missing information about the relationships of some of the group members at that moment in time. However, the technique described does not necessarily require the identities of all individuals to be known, as long as the subset that is sampled within a scan is the set of individuals that is *always* recorded. For example, Schreier & Swedell [26] collected nearest-neighbour identities of leader males within hamadryas baboon, *Papio hamadryas*, groupings using sequential scan samples, recording association between only these individuals without considering closer baboons who were not leader males. It could also be the case that some individuals may be absent or simply unidentifiable during one or more of the sampling scans. In this case, the clustering metric would be biased to the same degree as any other association metric, and should deliver similar biased results (albeit with the reduced number of samples described in §4).

The construction of a measure similar to nearest-neighbour clusters has been implicitly used in some field studies where subgroup membership is recorded, rather than nearest-neighbour identities. For example, Ramos-Fernández *et al*. [27] describe an observational chain-rule technique for use in the field which yields a similar division of individuals into subgroups, whereas Le Pendu *et al*. [28] and Hirotani [29] place individuals into subgroups based on a maximum distance between individuals, and Aureli *et al*. [30] use inter-individual distances as a means of computing subgroup membership. However, techniques like these where clusters are identified using some predefined spatial metric may lose information about subtler long-distance associations between individuals, which would be avoided if the metric described in this paper were used. Similarly, some studies consider an arbitrary cut-off distance for identifying a neighbour (e.g. [20,31–33]). Individuals closer than this cut-off are counted as neighbours, and those that are further are not. Again, subtle associations may be lost if we include an arbitrary cut-off, and even motivating a cut-off using a well-motivated biological reason (such as the feeding distance argument used by White & Burgman [33]) may miss associations that are occurring for different biological reasons.

The technique described here can be used to generate a matrix of associations between identifiable individuals that is demonstrably faster than simply considering just the counts of nearest-neighbour association. Once generated, these summary metrics still need to be processed to give meaningful comparable measures of association. For examples of how analyses can be conducted, I recommend the studies described in Henzi *et al*. [6] and Ramos-Fernández [27], and the general recommendations given in Whitehead [1] and Whitehead & Dufault [4]. A ‘sociability index’ based on simultaneous nearest-neighbour identification is proposed in Sibbald *et al*. [10] and further extended in Della-Rossa *et al*. [34], which can use both the simple nearest-neighbour identity metric and an extended version that considers second- and third-closest neighbours. Finally, as with any behavioural data, a suitable number of observations of dyadic associations between individuals is required if statistical tests are intended for the data collected: the technique described here may allow you do more with a sparse dataset, but cannot cover cases where too little has been collected, and Whitehead [35] gives recommendations for how to assess the precision and power of datasets.

## Supplementary Material

PROCESSING CODE The file called ‘processing_code.txt’ is annotated C++ code for analysing data, with details of how to format the data. The code presented will read comma-delimited (.csv) files formatted in MS-DOS (such as those saved by Microsoft Excel) - changes may need to be made to the input specification if you intend to use comma-delimited files formatted for a different operating system. NETLOGO SIMULATION CODE The files named ‘less_similar.nlogo.gz’, ‘most_similar.nlogo.gz’ and ‘random.nlogo.gz’ are zipped files containing three Netlogo files corresponding to the three models described in the appendix. Opening each of these in Netlogo 5.0.5 and hitting the ‘run-set’ button in the interface will generate a single text file called ‘resultset’txt’, and the if the default parameters given when the file is opened are nt changed, this will automatically generate the 100 simulations analysed in the manuscript, each separated with a line of asterisks.

## Supplementary Material

less_similar.nlogo

## Supplementary Material

most_similar.nlogo

## Supplementary Material

random.nlogo

## Data accessibility

Full source code to reproduce all data included in this study is included as supplementary information.

## Appendix A. Details of NetLogo models used for generating sample datasets

At the start of each simulation, an empty environment was created, measuring 201×201 cells of unit length arranged on a torus (a wrapped square where individuals moving off the left edge would appear on the right, and moving off the bottom edge will reappear at the top). Twenty-five individuals had an ordering hierarchy imposed on them, by numbering them sequentially between 1 and 25. These numbered individuals were then randomly placed at the centre of randomly selected cells, such that no cell contained more than one individual.

Over a series of consecutive timesteps (5 000 000 timesteps in the simulations, although the figures only show the results relating to the earlier observations for the sake of clarity), individuals moved within the environment: a timestep consisted of moving each of the individuals once, where the order in which individuals were picked was randomized at the beginning of each timestep. In a timestep, each individual chose to do one of two actions, where the choice of action was determined by generating a random number. Ninety per cent of the time, an individual moved one unit away from its current location in a random selected direction, where all possible headings were equally likely to be selected. Ten per cent of the time, the focal individual chose another individual within the group, turned to face towards it and then moved forwards one step. The identity of the individual chosen to move towards differed between the three models:

1. *Most similar hierarchy attraction*. The focal individual randomly chose one of the two individuals on either side of it within the ordering hierarchy (so individual 6 would choose between individual 5 or individual 7). Individual 1 was only able to choose individual 2 to orient towards, and individual 25 was only able to choose individual 24.
2. *Less similar hierarchy attraction*. The focal individual randomly chose one of the two individuals below it or two individuals above it within the ordering hierarchy (so individual 6 would choose between individuals 4, 5, 7 or 8). At the ends of the hierarchy, individuals may have been constrained by the choices available to them: therefore, individual 1 could only choose between individuals 2 or 3, and individual 24 could only choose between individuals 22, 23 and 25.
3. *Random attraction*. The focal individual chosen was randomly selected from all the other individuals in the group.

Within each model, nearest-neighbour identities were recorded for each individual on every 500th timestep.
